# The impact of COVID-19 pandemic on depression and anxiety symptoms: Findings from the United Arab Emirates Healthy Future (UAEHFS) cohort study

**DOI:** 10.1371/journal.pone.0277684

**Published:** 2022-11-16

**Authors:** Manal Al Blooshi, Tamadher Al Ameri, Maryam Al Marri, Amar Ahmad, Andrea Leinberger-Jabari, Abdishakur Abdulle, Manal Taimah, Thekra Al Zaabi, Khaloud Al Remeithi, Ayesha Al Hosani, Scott Sherman, Raghib Ali

**Affiliations:** 1 New York University Abu Dhabi, Abu Dhabi, United Arab Emirates; 2 New York University School of Medicine, New York, New York, United States of America; Zagazig University Faculty of Human Medicine, EGYPT

## Abstract

**Background:**

Significant concerns about mental health were raised during the COVID-19 pandemic. We investigated the prevalence of depression and anxiety symptoms among the participants of the United Arab Emirates Healthy Future Study (UAEHFS); a national cohort study. We further explored the change in the prevalence of depression symptoms among those with comparable pre-pandemic data.

**Methods:**

A sample of UAEHFS participants were invited to complete a COVID-19 online questionnaire during the first wave of the pandemic. Depression and anxiety symptoms were assessed using the Patient Health Questionnaire Depression Scale (PHQ-8) and the Generalized Anxiety Disorder-7 Scale (GAD-7) respectively. Unpaired analyses were done to examine the effect of COVID-19 on depression and anxiety symptoms during the pandemic. Paired analysis was conducted to examine the change in depression symptoms.

**Results:**

During the pandemic, we reported a prevalence of 32.8% (95% CI: 27.0, 39.1) for depression and 26.4% (95% CI: 21.0, 32.6) for anxiety symptoms. Younger people reported higher levels of depression (40.4%) and anxiety (34.5%) symptoms. Females reported higher levels of depression (36.5%) and anxiety (32.7%) symptoms. In paired analysis, the prevalence of depression symptoms during the pandemic was 34% (95% CI: 26.5, 42.4) compared to 29.9% (95% CI: 22.7, 38.1) before the pandemic. No statistically significant difference was observed, p-value = 0.440. Adjusted multivariate logistic regression models for PHQ-8 and GAD-7 during the pandemic showed that participants, who were experiencing flu-like symptoms, had higher odds of reporting depression symptoms compared to those without symptoms. Additionally, age was significantly negatively associated with anxiety symptoms.

**Conclusions:**

Overall, we found that depression and anxiety symptoms were more prevalent among young people and females. However, we did not find a significant change in the prevalence of depression symptoms among those with comparable pre-pandemic data. Identifying vulnerable groups and understanding trajectories through longitudinal studies would help with planning for effective mental health interventions for the current and future pandemics.

## 1. Introduction

Coronavirus disease 2019 (COVID-19) is an infectious illness caused by Acute Respiratory Syndrome Coronavirus-2 (SARS-CoV-2). On March 11^th^, 2020, the World Health Organization declared COVID-19 as a global pandemic [1]. As of October 19, 2022, the total confirmed cases of COVID-19 worldwide had reached 630 million, of which, 1,033,458 (10.25% of the population) were reported in the United Arab Emirates (UAE) [2]. In response to the pandemic, precautionary measures were implemented worldwide to curb the spread of the virus [3, 4]. the UAE government have adopted a range of these measures including: quarantines and lockdowns, travel restrictions, school and universities closure, and suspension of mass gatherings [5].

In any pandemic, it is common for individuals to feel confused, worried, and stressed [6]. Several stressors may affect individuals directly or indirectly during crisis these include: the risk of being infected and or infecting others, feelings of helplessness, boredom and frustration, fear of being socially excluded and economic implications [6, 7]. Findings from studies of pervious epidemics revealed a wide range of psychological impacts including depression and anxiety [8, 9]. Finding from studies examining the impact of COVID-19 on mental health echo those findings in both general and clinical populations [3, 10, 11]. Evidence from around the world have shown elevated levels of mental health problems including: fear [4, 10], emotional distress [12], sleep problems [13], anxiety, and depression [13, 14]. Similarly, several studies were conducted in the Gulf region and findings reported changes in levels of depression, anxiety and stress among studied populations [15–21].

In the UAE, multiple studies investigated the effect of the COVID-19 pandemic on depression and anxiety. A study conducted at the early stages of the pandemic assessed levels of anxiety and depression symptoms among adults to understand the impact of COVID-19 and its psychosocial correlates [22]. The psychological impact of the COVID-19 pandemic on adults and children was explored in another cross-sectional study [23]. Additionally, the impact of COVID-19 societal lockdown measures was assessed among UAE residents’ mental health [24]. The reviewed cross-sectional studies showed a marked increase in anxiety and depression levels in some demographic sub-groups. Students, young people and females had higher levels of depression and anxiety symptoms.

Several longitudinal studies examined changes in mental health by comparing pre- and during-COVID-19 data. A study conducted in the United States (US) reported elevated rates of psychological distress among US adult in April 2020 compared to those in 2018 [25]. Moreover, a study in the United Kingdom (UK) examined mental distress found a significant increase in the mean scores between 2018–2019 and April 2020 [26]. A Dutch study focused on depression and anxiety symptoms, analyzed data collected over three time points: November 2019, March 2020 and June 2020, found a non-significant increase in the prevalence of symptoms levels between November 2019 and March 2020. However, a significant reduction in the prevalence was observed between March 2020 and June 2020 [27]. Similarly, a Canadian study on young adults reported that depression and anxiety symptoms did not significantly change during the first wave of the pandemic [28].

To date, evidence from the UAE has been limited by use of cross-sectional design and a lack of comparable pre-COVID-19 data. In the present study we used data from a longitudinal study, namely the UAE Healthy Future Study (UAEHFS), to assess the impact of COVID-19 pandemic on depression and anxiety symptoms during the first wave of the pandemic approximately the tenth weeks after the national lockdown. We further explored the associated risk factors with depression and anxiety symptoms during the pandemic; and examined the change in the prevalence of depression symptoms among those with comparable pre-pandemic data.

## 2. Material and methods

### 2.1 Study design and participants

The UAEHFS is an ongoing, longitudinal cohort study that began in 2016 with the aim to identify the causes of chronic disease among UAE nationals; mainly diabetes, obesity and cardiovascular diseases. The participation consists of completing a detailed questionnaire on: socio-demographics and occupation, lifestyle exposures (including smoking, physical activity and diet), early life exposures, environmental exposures, psychological state, family history of illness, medical history and general health; the details of which have been published elsewhere [29].

**Fig 1:**
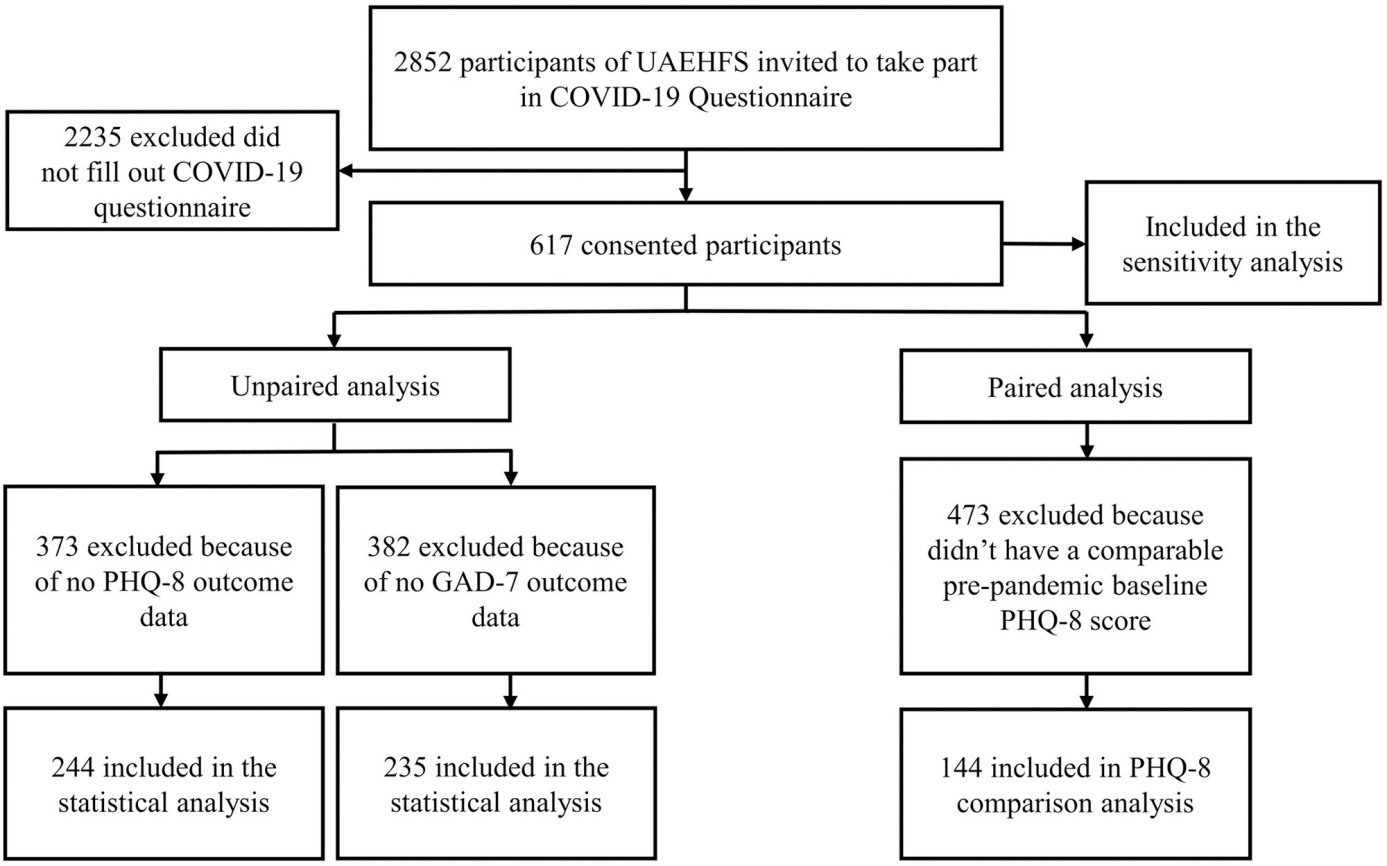
Flow chart showing selection into the study.

An online COVID-19 questionnaire was designed during the initial peak of the COVID-19 pandemic and distributed on the 10th week of the national lockdown. Existing UAEHFS participants who aged between 18 and 40 at enrolment were invited to participate in this study. Invitations and reminders were sent to participants via email (Fig 1). The survey was administrated via Voxco Survey Platform [30]

The study protocol was approved by the Research Ethics Committee of Abu Dhabi. Health Research and Technology Committee, reference number DOH/HQD/2020/516. Online written consent was given by participants prior to beginning of the questionnaire.

### 2.2 Procedures and measures

Data used in this study were extracted from the UAEHFS main questionnaire and the COVID-19 questionnaire. Both questionnaires were translated into Arabic and back into English for linguistic validation [29]. For the Personal Health Questionnaire Depression Scale (PHQ-8) and General Anxiety Disorder Scale (GAD-7), they were already available in Arabic and English [31]. Participants had the option to choose their preferred language (English or Arabic).

#### 2.2.1 Demographics

Some demographic variables were extracted from the main UAEHFS questionnaire. These included: gender (female, male), age (18–24, 25–29, 30–34, 35–39, 40–44), education level (lower than university, university, postgraduate), and employment status (employed, Student, unemployed). The data extracted from the COVID-19 questionnaire included marital status (single, married, divorced/widowed), and whether they were working remotely during the pandemic (yes, no). The COVID-19 questionnaire asked whether they have been told by a doctor that they suffer from any chronic health conditions (yes, no) or experienced flu-like symptoms (yes, no). Participants were asked if they knew someone infected with the virus (yes, no).

#### 2.2.2 Pandemic worries

To assess pandemic worries, participants were asked to rate how worried they were on five questions which were previously published [32]: “I am worried about getting sick myself”, “I am worried that someone close to me will get sick”, “I am worried that it will take a long time before I can resume my normal daily life”, “I am worried that I or my family will be in serious financial trouble” and “I am worried that I cannot see my family and friends”. Participants responded on a 5-point Likert scale from “1: extremely worried” to “5: not worried at all”.

#### 2.2.3 Depression symptoms (before and during the pandemic)

To indicate the prevalence of depression symptoms, we used the Patient Health Questionnaire-8 (PHQ-8). The PHQ-8 is an instrument that consists of eight questions assessing depression symptoms over the past two weeks on administration of the assessment [33]. Scoring intervals range from 0 to 24 and are obtained by summing the responses to each question. A score from 0 to 9 represents the absence of depression symptoms. A total score of 10 and above indicate the presence of depression symptoms. For the current analysis, a cut-off value of 10 was used to dichotomize into no-depression symptoms (total PHQ-8<10) versus depression symptoms (≥ 10) [33]. The estimated Cronbach alpha for the PHQ-8 showed good internal reliability before (alpha = 0.868) and during (alpha = 0.898) the pandemic.

#### 2.2.4 Anxiety symptoms (during the pandemic)

To estimate the prevalence of anxiety symptoms, we used the Generalized Anxiety Disorder-7 (GAD-7) [34]. The index has seven items that measure worry and anxiety symptoms during the past two weeks of administration of assessment. It is scored on a four-point Likert scale (0–3) with a total score summing the responses to each item ranging from 0–21. The higher the score, the greater anxiety severity; a score beyond 10 is considered a cut off for anxiety symptoms [35]. For the current analysis, a binary GAD-7 score was computed by dichotomizing into no-anxiety symptoms (total GAD-7<10) versus anxiety symptoms (≥ 10) [34]. An excellent Cronbach alpha was estimated between the items for GAD-7 (alpha = 0.906).

### 2.3 Statistical analysis

All applied statistical tests were two-sided and p-values less than 0.05 were considered as statistically significant. No adjustments for multiple comparisons were made. Statistical analysis was conducted using r version 4.0.2 [36]. The statistical methods were documented in a pre-specified statistical analysis plan to minimize bias in the results. In both questionnaires, each question had two additional answer options: “Prefer not to answer” and “I don’t know”. These options were categorized as “unknown”.

In the paired analysis, categorical data was presented in frequencies and percentages. Missing values in the variables were categorized as “unknown”. Chi-square (χ2) tests were performed to explore the associations between participant demographics, general health, pandemic worries with depression and anxiety symptoms, respectively. Correspondently, p-values were computed using 2000 Monte Carlo simulation. For each outcome, such as the dichotomized PHQ-8 and GAD-7, a multivariate logistic regression model was performed respectively. The predictors were age, gender, marital status, education level, employment status, working remotely, presence of health condition, experiencing flu-like symptoms and knowing someone infected with COVID-19. Odds ratios (ORs) and confidence intervals (95% CI) were computed, and corresponding z-values and P-values were reported. The estimated area under the curve (AUC) corresponding 95% CI for each multivariate logistic regression model was calculated to assess the model performance. In the paired analysis, exact McNemar test was performed to compare the PHQ-8 pre and during COVID-19 pandemic. A sensitivity analysis was performed with 200 multiple imputations. Rubin´s Rules were used to pool the ORs of the McNemar test.

## 3. Results

Out of 617 consented participants, 244 (39.5%) and 235 (38.1%), who completed the questionnaire for PHQ-8 and GAD-7 scores, respectively, were included in the unpaired analysis (Fig 1). The median age of the study participant was 28 years (Interquartile range (IQ): 22–35).

Table 1 summarizes the frequency (percentages) of each categorical variable presented by the dichotomized PHQ-8 and GAD-7 categories during the pandemic. Participants were more likely to be females, aged 19 to 24 years, single, hold a university degree and employed. Around 25% indicated that they were working remotely due to the pandemic. Almost 36% reported having at health condition, and nearly 30% were experiencing flu-like symptoms. The majority of participants reported knowing someone infected with COVID-19 (Table 1).

**Table 1:** Demographic characteristics by the dichotomized PHQ-8 score (PHQ-8>10) and dichotomized anxiety score (GAD-7 >10) during the pandemic.

Variable	Category	Depression			Anxiety		
		dichotomized PHQ-8 score			dichotomized GAD-7 score		
		(n-244)			(n = 235)		
		n(%)	Yes (n)%	P-value	n(%)	Yes (n)%	P-value
			PHQ-8>10			GAD-7>10	
**Age**	18–24	114 (47)	46 (40.4)	0.137	110 (47)	38 (34.5)	0.085
	25–29	38 (16)	12 (31.6)		37 (16)	8 (21.6)	
	30–34	46 (19)	10 (21.7)		43 (18)	9 (20.9)	
	35–39	40 (16)	10 (25)		39 (17)	7 (17.9)	
	40–44	6 (3)	2 (33.3)		6 (3)	0 (0)	
**Gender**	Female	167 (68)	61 (36.5)	0.078	159 (68)	52 (32.7)	0.002*
	Male	77 (32)	19 (24.7)		76 (32)	10 (13.2)	
**Marital Status**	Single	164 (67)	62 (37.8)	0.039*	161 (69)	48 (29.8)	0.017*
	Divorced/Widowed	9 (4)	3 (33.3)		8 (3)	4 (50)	
	Married	71 (29)	15 (21.1)		66 (28)	10 (15.2)	
**Education**	University	107 (44)	37 (34.6)	0.699	102 (43)	26 (25.5)	0.634
	Lower than University	95 (39)	27 (28.4)		92 (39)	23 (25)	
	Postgraduate	11 (5)	4 (36.4)		10 (4)	4 (40)	
	Unknown**	31 (13)	12 (38.7)		31 (13)	9 (29)	
**Employment Status** ***	Employed	102 (42)	28 (27.5)	0.085	97 (41)	23 (23.7)	0.007*
	Student	78 (32)	34 (43.6)		75 (32)	30 (40)	
	Unemployed	42 (17)	14 (33.3)		41 (17)	6 (14.6)	
	unknown**	22 (9)	4 (18.2)		22 (9)	3 (13.6)	
**Working Remotely**	Yes	62 (25)	11 (17.7)	0.0154*	61 (26)	11 (18)	0.078
	No	48 (20)	18 (37.5)		44 (19)	14 (31.8)	
	Students & unemployed	120 (49)	48 (40)		116 (49)	36 (31%)	
	Unknown**	14 (6)	3 (21)		14 (6)	1 (7%)	
**Health Condition**	No	157 (64)	49 (31.2)	<0.001*	150 (64)	43 (28.7)	<0.001*
	Yes	87 (36)	31 (35.6)		85 (36)	19 (22.4)	
**Experiencing flu-like symptom**	No	171 (70)	47 (27.5)	<0.001*	166 (71)	34 (20.5)	<0.001*
	Yes	73 (30)	33 (45.2)		69 (29)	28 (40.6)	
**Knowing someone infected with COVID-19**	No	89 (37)	33 (37.1)	0.387	85 (36)	24 (28.2)	0.736
	Unknown**	12 (5)	3 (25)		12 (5)	4 (33.3)	
	Yes	143 (59)	44 (30.8)		138 (59)	34 (24.6)	

* Significant at p < 0.05

**"Preferred not answer" or "I don’t know" values were categorized as "unknown"

*** measured pre-pandemic

### 3.1 Depression and anxiety symptoms during the pandemic

A strong Spearman rank correlation was found between the total PHQ-8 score and GAD-7-score 0.757 (95%CI: 0.692, 0.810). The prevalence of depression during the pandemic was 32.8% (95% CI: 27.0, 39.1). Depression was more prevalent among participants who were single (37.5%), among students (43.6%), those who were reporting to workplaces (37.5%), had a health condition (35.6%) and those who had experienced flu-like symptoms (45.2%), p value <0.05 (Table 1). Depression was higher in females (36.5%) and participants between the ages of 18 and 24 years (40.4%), but differences were not significant.

The prevalence of anxiety during the pandemic was 26.4% (95% CI: 21.0, 32.6). Overall, female participants (32.7%) were more anxious than males (13.2%). Students showed significant differences in anxiety compared to employed participants; 40% and 23.7% respectively. Participants who had a health condition and were experiencing flu-like symptoms were more anxious (40.6%). However, anxiety was higher among participants with higher levels of education but differences were not significant. (Table 1).

### 3.2 Pandemic worries

Table 2 shows that participants were mostly worried about “someone close to them will get sick” and “that it will take a long time before they can resume a normal daily life”. For depression, participants who were extremely worried that “someone close to them will get sick” were significantly more likely to report depression (48.8%) compared to those who were not worried at all (17.6%). Similarly, participants who were extremely worried that it will take a long time before they can resume a normal daily life were significantly more likely to report depression (41.7%) compared to those who were not worried at all (15.4%). Significantly higher percentages of anxiety were reported among participants who were extremely worried that: someone close to them will get sick (45.8%), it will take a long time before they can resume a normal daily life (46.2%), they will be in serious financial trouble (44.7%), they will not be able to see their family or friends (42.9%).

**Table 2:** Pandemic worries by the dichotomized PHQ-8 score (PHQ-8>10) and dichotomized anxiety score (GAD-7 >10) during the pandemic.

Variable	Category	Depression			Anxiety		
		dichotomized PHQ-8 score			dichotomized GAD-7 score		
		(n-244)			(n = 235)		
		n(%)	Yes (n)%	P-value	n(%)	Yes (n)%	P-value
			PHQ-8>10			GAD-7>10	
**I am worried about getting sick myself**	Extremely worried	21 (9)	10 (47.6)	0.349	19 (8)	9 (47.4)	0.19
	Very worried	29 (12)	11 (37.9)		28 (12)	8 (28.6)	
	Somewhat worried	62 (25)	17 (27.4)		60 (26)	13 (21.7)	
	Little worried	71 (29)	20 (28.2)		69 (29)	15 (21.7)	
	Not worried at all	55 (23)	20 (36.4)		54 (23)	16 (29.6)	
	Unknown**	6 (2)	2 (33.3)		5 (2)	1 (20)	
**I am worried that someone close to me will get sick**	Extremely worried	60 (25)	25 (41.7)	**0.025**	59 (25)	27 (45.8)	**<0.001** *
	Very worried	62 (25)	21 (33.9)		59 (25)	13 (22)	
	Somewhat worried	52 (21)	21 (40.4)		50 (21)	13 (26)	
	Little worried	40 (16)	7 (17.5)		39 (17)	5 (12.8)	
	Not worried at all	26 (11)	4 (15.4)		25 (11)	3 (12)	
	Unknown**	4 (2)	2 (50)		3 (1)	1 (33.3)	
**I am worried that it will take a long time before I can resume my normal daily life**	Extremely worried	41 (17)	20 (48.8)	**0.017**	39 (17)	18 (46.2)	**0.022**
	Very worried	49 (20)	19 (38.8)		47 (20)	14 (29.8)	
	Somewhat worried	64 (26)	21 (32.8)		63 (27)	13 (20.6)	
	Little worried	47 (19)	10 (21.3)		46 (20)	9 (19.6)	
	Not worried at all	34 (14)	6 (17.6)		32 (14)	5 (15.6)	
	Unknown**	9 (4)	4 (44.4)		8 (3)	3 (37.5)	
**I am worried that I or my family will be in serious financial trouble**	Extremely worried	39 (16)	18 (46.2)	0.3	38 (16)	17 (44.7)	**0.051**
	Very worried	26 (11)	8 (30.8)		26 (11)	7 (26.9)	
	Somewhat worried	30 (12)	9 (30)		27 (11)	4 (14.8)	
	Little worried	35 (14)	12 (34.3)		33 (14)	9 (27.3)	
	Not worried at all	98 (40)	26 (26.5)		96 (41)	21 (21.9)	
	Unknown**	16 (7)	7 (43.8)		15 (6)	4 (26.7)	
**I am worried that I cannot see my family and friends**	Extremely worried	36 (15)	17 (47.2)	0.352	35 (15)	15 (42.9)	**0.045**
	Very worried	47 (19)	14 (29.8)		45 (19)	10 (22.2)	
	Somewhat worried	45 (18)	15 (33.3)		44 (19)	15 (34.1)	
	Little worried	47 (19)	15 (31.9)		47 (20)	7 (14.9)	
	Not worried at all	59 (24)	16 (27.1)		55 (23)	13 (23.6)	
	Unknown**	10 (4)	3 (30)		9 (4)	2 (22.2)	

* Significant at p < 0.05

**"Preferred not answer" or "I don’t know" values were categorized as "unknown"

Table 3 shows the results of multivariate logistic regression models with the dichotomized PHQ-8 score GAD-7 score as outcomes. In both models, the effects of age, gender, marital status, education level, employment status, remote work, presence of health condition, experiencing flu-like symptoms and knowing someone infected with COVID-19 were assessed. Estimated ORs and corresponding 95% CIs as well as Wald’s z values (and p-values) are illustrated in Table 3. In model A, participants who were experiencing flu-like symptoms had 2.12 times higher odds of reporting depression compared to those without symptoms. In model B, age was statistically significant negatively associated with anxiety OR of 0.9 (95%CI: 0.82, 0.99), P value = 0.03. Participants experiencing flu-like symptoms had 2.2 times higher odds of reporting anxiety than those without symptoms. The estimated area under the curve (AUC) corresponding 95% CI for model A is 0.696 (95% CI: 0.6275–0.7583) (500 stratified bootstrap replicates) and for model B 0.763 (95% CI: 0.6961–0.8288) (500 stratified bootstrap replicates), which shows a good models’ performance.

**Table 3:** Multivariate logistic regression models with dichotomized PHQ-8 and GAD-7 as outcomes.

	Model A (PHQ-8)		Model B (GAD-7)	
	dichotomized PHQ-8 score		dichotomized GAD-7 score	
Variable	OR (95% CI)	z value (p)	OR (95% CI)	z value (p)
Age	0.998 (0.93, 1.07)	-0.05 (0.96)	0.9 (0.82, 0.99)	-2.22 (0.03) *
gender = Male	1.104 (0.53, 2.28)	0.27 (0.79)	0.439 (0.18, 1.06)	-1.82 (0.07)
Marital Status = Divorced/Widowed	0.635 (0.13, 3.19)	-0.55 (0.58)	4.82 (0.79, 29.44)	1.7 (0.09)
Marital Status = Married	0.552 (0.24, 1.25)	-1.43 (0.15)	0.76 (0.29, 2)	-0.56 (0.58)
Education = Lower than University	0.682 (0.34, 1.36)	-1.09 (0.28)	0.833 (0.38, 1.81)	-0.46 (0.64)
Education = Postgraduate	1.486 (0.36, 6.08)	0.55 (0.58)	3.01 (0.66, 13.79)	1.42 (0.16)
Education = unknown	0.884 (0.36, 2.16)	-0.27 (0.79)	0.769 (0.27, 2.18)	-0.49 (0.62)
Employment Status = student	1.879 (0.33, 10.63)	0.71 (0.48)	3.423 (0.49, 24.09)	1.24 (0.22)
Employment Status = unemployed	1.365 (0.26, 7.07)	0.37 (0.71)	1.501 (0.23, 9.88)	0.42 (0.67)
Employment Status = unknown	0.721 (0.15, 3.39)	-0.41 (0.68)	0.782 (0.14, 4.34)	-0.28 (0.78)
Heath condition = Yes	1.242 (0.66, 2.33)	0.67 (0.5)	0.831 (0.4, 1.75)	-0.49 (0.63)
Flu-like symptoms = Yes	2.12 (1.13, 3.99)	2.33 (0.02)*	2.2 (1.1, 4.4)	2.23 (0.03)*
Knowing someone infected = unknown	0.549 (0.13, 2.41)	-0.79 (0.43)	2.65 (0.51, 13.65)	1.17 (0.24)
Knowing someone infected = Yes	0.633 (0.34, 1.17)	-1.45 (0.15)	0.657 (0.33, 1.32)	-1.18 (0.24)
Remote work = No	2.431 (0.92, 6.39)	1.8 (0.07)	1.302 (0.44, 3.83)	0.48 (0.63)
Remote work = unknown	1.531 (0.32, 7.35)	0.53 (0.59)	0.182 (0.03, 1.11)	-1.85 (0.06)

### 3.3 Depression symptoms before and during the pandemic

A comparison analysis was performed for 144 participants with a comparable pre-pandemic baseline PHQ-8 data (Fig 1). The prevalence of depression before and during pandemic using the dichotomized PHQ-8 was 29.9% (95% CI: 22.7, 38.1) and 34% (95% CI: 0.265, 0.424) respectively. No statistically significant difference was observed, OR = 1.33 (95% CI: 0.69, 2.61), exact McNemar test’s p-value = 0.441. The result of the sensitivity analysis using 200 multiple imputations gave an OR = 1.77 (95% CI: 1.23, 2.52). S1 Fig shows the estimated ORs with corresponding 95% CIs from the omitted dataset and 200 imputed datasets.

## 4. Discussion

Using longitudinal data from the UAEHFS, this study is one of the few studies that explored the impact of COVID-19 pandemic on depression and anxiety symptoms among the youthful population of the UAE (the median age in the UAE is 32.6 years) [37]. COVID-19 related data was collected during the first wave of the pandemic. While, pre-COVID-19 data was collected approximately two years before the pandemic from the same study sample. We explored the prevalence of depression and anxiety symptoms during COVID-19 pandemic and assessed the change in depression symptoms pre- and during-COVID-19 pandemic.

The prevalence of depression and anxiety symptoms have previously been explored among the population of the UAE during the COVID-19 pandemic. In our study, the prevalence of depression and anxiety during the pandemic were (32.8%) and (26.4%) respectively. Both prevalence were lower than those was reported by [22] of 58.4% and 55.7% for depression and anxiety, respectively. The different prevalence of depression and anxiety could be explained by differences in the timing of studying the associations during the lockdown at the time of the first COVID-19 peak. Additionally, the sample composition was considerably different to our sample. Thomas and his colleagues used a larger sample size, which constituted of 65 different nationalities [22].

We found that during the COVID-19 pandemic, depression and anxiety symptoms were more prevalent among females, younger population age (19–25) and students, as well as participants who reported to work. Furthermore, participants who knew someone with COVID-19 or had flu like symptoms were more likely to report depression symptoms. Marital status showed an interesting correlation to the outcomes of interest. For instance, being married was protective against depression and anxiety. However, being divorced or widowed was protective against depression, but a risk factor to develop anxiety symptoms. In addition, a strong correlation was found between PHQ-8 score and GAD-7-score that reflects the comorbidity of these two conditions.

The higher prevalence of depression and anxiety symptoms during the COVID-19 pandemic among females is consistent with findings from other studies in the UAE [22–24], and globally [13, 38–40]. This could be explained by a social role expectation of women that they are seen as main caregivers, which can be, stress inducing. Moreover, during COVID-19 pandemic, most caring mothers had extra effort exerted at home watching their children remotely schooling on top of their jobs and family responsibilities [23, 24].

Though not statistically significant, the prevalence of depression and anxiety symptoms varied among different age groups. Aligned with findings from other studies in the UAE, and globally, youngest age group (18–24) years had the highest prevalence of depression and anxiety symptoms compared to other age groups. This finding is particularly significant for the UAE, which has a relatively youthful population. The increase in depression and anxiety symptoms in the younger age group can be explained by social factors. We found that the prevalence of depression and anxiety were higher among students and majority of this age group are (18–24) students. Study exploring this further in the UAE, reported that students above the age of 20 had significantly more anxiety and depression in comparison to students below 20 years of age [41]. Students’ lifestyle is known to be stressful and under the pandemic circumstances, students had to adopt a new online distant-learning, which may have created uncertainty regarding their education and future [42]. Additionally, distance learning may have exacerbated pre-existing stress levels and altered their motivation to study, increased level of pressure to learn independently and reduced student’s ability to use typical coping strategies and daily routines [43]. We found that there was a significant difference across marital status categories in relation to the outcomes; with being single scoring the highest. The results are contradicting, what was found in Kuwait and Saudi Arabia that marital status did not have any influence on anxiety levels of the respondents [16, 17]. However, in our study we found being divorced/ widowed was protective against depression, but increased the risk of anxiety by almost four times.

In the univariate analysis, working remotely was associated with lower anxiety and significant lower depression symptoms. This could be due to the reduction of stress-related to being exposed to coronavirus infection. Removing self from environments that are perceived to be risky can be protective against depression and anxiety symptoms. On the other hand, in the multivariate logistic regression analyses, reporting to work increased the risk of depression and anxiety. Having a flu-like symptom was associated with a significant increase in the odds of having depression and anxiety symptoms, as the perceived risk of being COVID positive, can elevate psychological symptoms due to an intense emotional response. Moreover, having a flu-like symptoms means that the individual is at risk of being quarantined, which induces negative emotions such as fear and loneliness [44]. Furthermore, pandemic worries such as being worried that someone close gets sick was significantly associated with elevated anxiety symptoms.

In the Middle East, which the UAE is part of, the communities are known to be collective in nature. One of the features of such communities is putting higher values to the groups’ wants and needs upon the individual’s [45]. Shekriladze and his colleagues reported that collectivism could induce stress via transferable emotions from family and friend to the individual, leading to a collective emotional response to a problem or event [46]. Psychologically, the susceptibility to depression and anxiety plays a major role in coping with the pandemic worries. For instance, individual patterns of thinking, feelings, behaviors and emotions determines personal perceptions on the pandemic and its consequences and threats [47, 48]. Moreover, people with high neuroticism tend to experience more intense negative emotions, such as, fear, anger, and irritability during stressful events [49, 50]. With the long duration of the pandemic and chronic poor coping mechanisms combined with intense emotional reactivity; individuals with high neuroticism tend to be at higher risk of developing depression and anxiety [48].

A secondary aim of the study was to explore the change in depression symptoms relative to pre-pandemic measure. We were able to compare the depression symptoms for 144 participants using data collected before and during the pandemic. For this group, the prevalence of depression symptoms before the pandemic was 29.9% and it increased to 34% during the pandemic but was not statistically significant. This finding is consistent with those reported in the Canadian longitudinal study in regards to the change in the prevalence of depression symptoms [28]. Similar result was reported in the Dutch longitudinal study, where prevalence of depression increased non significantly during March 2020, but decreased to significantly in June 2020 [27]. On the other hand, significant changes in mental health were reported in the UK population [26, 51]. A study investigating the trajectories of anxiety and depression symptoms in the UK reported that highest levels of symptoms occurred in the early stages of the lockdown but declined across the 20 weeks following the start of the lockdown [51]. The inconsistences in findings across countries could be due to different COVID-19 restrictive measures, timing of undertaking the research, sample representativeness and use of different assessment instruments.

This study has its strengths and limitations. The main strength of this study lies in the use of longitudinal data from the first national cohort prospective study in the UAE from the same participants. Moreover, the use of standardized instruments of depression and anxiety is another strength of this study. On the other hand, we did not include any COVID-19 related instruments such as the fear of COVID-19 Scale [52]. Including such instruments would have enriched our study. A significant limitation of our study is the sample size, which is relatively small. This may have reduced the statistical power, particularly in the comparison analysis. Therefore, our results were interpreted in an exploratory manner. Additionally, the use of convenience sampling may introduce some selection biases in the present study. For instance, our study includes more females (68%) than males (32%). However, this percentage is lower than reported in UAE-based similar studies 75% in [24] and 85.6% in [22]. Missing values were omitted in the primary statistical analysis, which decreased the sample size and may have led to a false negative result in the main finding. The sensitivity analysis showed an increase in the value of the OR with a statistically significant result. Due to sample representativeness, findings from this study may not be generalizable to other subgroups in the UAE and beyond including elderly and children.

## 5. Conclusions

The current study provides a general picture of the impact of COVID-19 pandemic on mental health among the youthful population of the UAEHFS during the first wave of the pandemic. We found that the prevalence of depression and anxiety symptoms were highest among the youngest age groups and females. The prevalence of depression symptoms increased compared to before the pandemic for those with pre-pandemic measure. Our study adds to the growing literature on the impact of COVID-19 pandemic on mental health in the UAE. To get a fuller picture, further work is needed using longitudinal studies with larger representative sample and subsequent follow ups. This would help in exploring trajectories of mental health problems over time and identifying vulnerable groups at a wider level.

## Supporting information

S1 Fig Result of the sensitivity analysis using 200 multiple imputations. (PDF)
