# Supplementary material for: The impact of COVID-19 pandemic on depression and anxiety symptoms: Findings from the United Arab Emirates Healthy Future (UAEHFS) cohort study
Source: PLoS One. 2022 Nov 16;17(11):e0277684. doi: 10.1371/journal.pone.0277684 (PMC9668125; doi:10.1371/journal.pone.0277684)
Supplement: S1 Fig — (PDF) [file pone.0277684.s001.pdf]

S1 Fig. Result of the sensitivity analysis using 200 multiple imputations

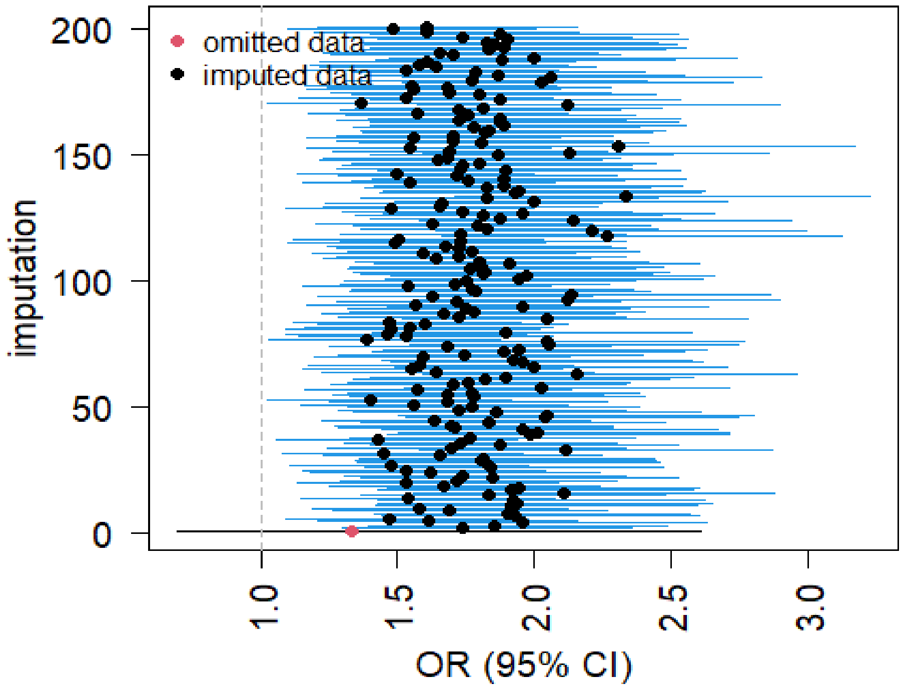

Estimated ORs with corresponding 95% CIs from the omitted dataset (red filled dot) and 200 imputed datasets (black filled dots). Dotted grey line shows the null hypothesis value (OR=1). All 95% CIs for the imputed datasets (blue solid lines) do not include the value of 1 which indicate statistically significant differences between the dichotomized PHQ-8 before and during COVID-19 pandemic. However the corresponding 95% CI for the completed case analysis (omitted data, black solid line), does include the value of 1 (not statistically significant). The pooled OR using Rubin's rules was 1.77 (95% CI: 1.23 2.52).
